# Intraocular human cytomegaloviruses of ocular diseases are distinct from those of viremia and are capable of escaping from innate and adaptive immunity by exploiting HLA-E-mediated peripheral and central tolerance

**DOI:** 10.3389/fimmu.2022.1008220

**Published:** 2022-10-19

**Authors:** Mariko Shirane, Nobuyo Yawata, Daisuke Motooka, Kensuke Shibata, Seik-Soon Khor, Yosuke Omae, Toshikatsu Kaburaki, Ryoji Yanai, Hisashi Mashimo, Satoshi Yamana, Takako Ito, Akira Hayashida, Yasuo Mori, Akihiko Numata, Yusuke Murakami, Kohta Fujiwara, Nobuyuki Ohguro, Mayumi Hosogai, Masato Akiyama, Eiichi Hasegawa, Michael Paley, Atsunobu Takeda, Katsumi Maenaka, Koichi Akashi, Wayne M. Yokoyama, Katsushi Tokunaga, Makoto Yawata, Koh-Hei Sonoda

**Affiliations:** ^1^ Department of Ophthalmology, Kyushu University, Fukuoka, Japan; ^2^ Department of Ocular Pathology and Imaging Science, Kyushu University, Fukuoka, Japan; ^3^ Ocular inflammation and Immunology, Singapore Eye Research Institute, Singapore, Singapore; ^4^ Ophthalmology and Visual Sciences Academic Clinical Program, Duke-NUS Medical School, Singapore, Singapore; ^5^ Department of Infection Metagenomics, Research Institute for Microbial Diseases, Osaka University, Osaka, Japan; ^6^ Integrated Frontier Research for Medical Science Division, Institute for Open and Transdisciplinary Research Initiatives (OTRI), Osaka University, Osaka, Japan; ^7^ Department of Microbiology and Immunology, Graduate School of Medicine, Yamaguchi University, Yamaguchi, Japan; ^8^ Department of Molecular Immunology, Research Institute for Microbial Diseases, Osaka University, Osaka, Japan; ^9^ Genome Medical Science Project, National Center for Global Health and Medicine, Tokyo, Japan; ^10^ Department of Ophthalmology, The University of Tokyo Hospital, Tokyo, Japan; ^11^ Department of Ophthalmology, Jichi Medical University Saitama Medical Center, Saitama, Japan; ^12^ Department of Ophthalmology, Yamaguchi University Graduate School of Medicine, Yamaguchi, Japan; ^13^ Department of Ophthalmology, Japan Community Health Care Organization Hospital, Osaka, Japan; ^14^ Department of Medicine and Biosystemic Science, Kyushu University Graduate School of Medical Science, Fukuoka, Japan; ^15^ Department of Ophthalmology, Gunma University Graduate School of Medicine, Gunma, Japan; ^16^ Department of Medicine, Washington University School of Medicine, St. Louis, MO, United States; ^17^ Center for Research and Education on Drug Discovery, Faculty of Pharmaceutical Sciences, Hokkaido University, Sapporo, Japan; ^18^ Laboratory of Biomolecular Science, Faculty of Pharmaceutical Sciences, Hokkaido University, Sapporo, Japan; ^19^ Global Station for Biosurfaces and Drug Discovery, Hokkaido University, Sapporo, Japan; ^20^ Bursky Center for Human Immunology and Immunotherapy Programs, Washington University, St. Louis, MO, United States; ^21^ Singapore Institute for Clinical Sciences (SICS), Agency for Science, Technology and Research, ASTAR, Singapore, Singapore; ^22^ Department of Pediatrics, Yong Loo Lin School of Medicine, National University of Singapore, Singapore, Singapore; ^23^ Department of Pediatrics, National University Health System, Singapore, Singapore; ^24^ Immunology Programme, Life Sciences Institute, National University of Singapore, Singapore, Singapore; ^25^ National University Singapore Medicine Immunology Translational Research Programme, National University of Singapore, Singapore, Singapore; ^26^ International Research Center for Medical Sciences, Kumamoto University, Kumamoto, Japan

**Keywords:** human cytomegalovirus, HLA-E, UL40, natural killer cells, HLA class I, NKG2A, CMV viremia, CMV retinitis

## Abstract

Human cytomegalovirus (HCMV) infections develop into CMV diseases that result in various forms of manifestations in local organs. CMV-retinitis is a form of CMV disease that develops in immunocompromised hosts with CMV-viremia after viruses in the peripheral circulation have entered the eye. In the HCMV genome, extensive diversification of the UL40 gene has produced peptide sequences that modulate NK cell effector functions when loaded onto HLA-E and are subsequently recognized by the NKG2A and NKG2C receptors. Notably, some HCMV strains carry UL40 genes that encode peptide sequences identical to the signal peptide sequences of specific HLA-A and HLA-C allotypes, which enables these CMV strains to escape HLA-E-restricted CD8^+^T cell responses. Variations in UL40 sequences have been studied mainly in the peripheral blood of CMV-viremia cases. In this study, we sought to investigate how ocular CMV disease develops from CMV infections. CMV gene sequences were compared between the intraocular fluids and peripheral blood of 77 clinical cases. UL40 signal peptide sequences were more diverse, and multiple sequences were typically present in CMV-viremia blood compared to intraocular fluid. Significantly stronger NK cell suppression was induced by UL40-derived peptides from intraocular HCMV compared to those identified only in peripheral blood. HCMV present in intraocular fluids were limited to those carrying a UL40 peptide sequence corresponding to the leader peptide sequence of the host’s HLA class I, while UL40-derived peptides from HCMV found only in the peripheral blood were disparate from any HLA class I allotype. Overall, our analyses of CMV-retinitis inferred that specific HCMV strains with UL40 signal sequences matching the host’s HLA signal peptide sequences were those that crossed the blood–ocular barrier to enter the intraocular space. UL40 peptide repertoires were the same in the intraocular fluids of all ocular CMV diseases, regardless of host immune status, implying that virus type is likely to be a common determinant in ocular CMV disease development. We thus propose a mechanism for ocular CMV disease development, in which particular HCMV types in the blood exploit peripheral and central HLA-E-mediated tolerance mechanisms and, thus, escape the antivirus responses of both innate and adaptive immunity.

## Introduction

Human cytomegalovirus (HCMV) latently infects 50-90% of the global population, and multiple subclinical reactivations occur over a host’s lifetime. HCMV reactivation manifests as CMV-viremia in up to 65% of immunocompromised hosts who have undergone hematopoietic stem cell transplantation; however, only 5-11% of CMV-viremia patients develop CMV-retinitis, a typical CMV disease resulting in severe visual impairment (1–6). Circulating leukocytes are major sites for latent HCMV infection, and the vascular endothelium is considered the primary site of virus entry into local tissues, including the eye (7–10). Therefore, immune responses at the vascular endothelium upon HCMV infection are of prime importance in the pathogenesis of ocular CMV diseases (10, 11).

HCMV is a diverse family of viruses that express various proteins involved in immune evasion (12–14). HCMV genome information is limited to strains isolated from peripheral blood and urine, and the differences between viruses in the peripheral blood and those in local infected tissues are underexplored (15).

CD8^+^ cytotoxic T cells and natural killer (NK) cells play central roles in controlling HCMV (12, 16). The HCMV glycoprotein US2 downregulates the cell-surface expression of the classical HLA class I molecules HLA-A and HLA-B, which enable the virus to escape conventional CD8^+^ T cells (17). However, downregulation of HLA class I has the potential to trigger innate immune responses through the “missing-self” response of NK cells (18–21). HCMV utilizes escape mechanisms that center on the “non-classical” HLA class I molecule HLA-E, which is recognized by the inhibitory and activating receptors NKG2A and NKG2C, respectively, and the T cell receptor. Because the expression of HLA-E is dependent on the loading of signal peptides from classical HLA class I, the downregulation of HLA-A and -B results in the downregulation of HLA-E as well, which in turn induces the missing-self response in NKG2A^+^ NK cells (18, 22). HCMV has evolved a notable strategy to maintain HLA-E expression levels and circumvent NK cell responses by supplying signal peptides encoded by the polymorphic virus UL40 gene (23). Of note, different UL40-derived signal peptides have distinct affinities for HLA-E and NKG2A, resulting in variable NK-cell inhibition (24–27). Furthermore, among the various UL40-derived signal peptides, three, V**M**APRTL**I**L, V**M**APRTL**V**L, and V**M**APRTL**L**L, are identical to signal peptides of the classical HLA class I subsets, which may result in viruses escaping from HLA-E-restricted T cells (28–31).

Over 30 HCMV UL40-encoding signal peptides have been reported from clinical isolates from CMV-viremia patients, and the polymorphisms were reportedly clustered in the signal peptide region (24, 32–35). Signatures of diversifying selection in this genomic region imply the occurrence of past and ongoing virus–host interactions (32). Until now, polymorphisms of UL40 peptides have been mainly studied using blood samples of CMV-viremia patients in Caucasian population groups. Moreover, how polymorphisms of UL40 are associated with the clinical manifestations of CMV diseases is not well understood. This study examined UL40 variants in the ocular fluids of ocular CMV disease patients and the blood of CMV-viremia patients in the Japanese population with the aim of determining their functional significance in ocular disease progression.

## Materials and methods

### Study participants

In this study, 77 patients with CMV cases, i.e., 29 CMV-viremia, 14 CMV-retinitis, 27 CMV-anterior uveitis (CMV-AU), and 7 CMV-chronic retinal necrosis (CMV-CRN) cases, were recruited at Kyushu University Hospital; University of Tokyo Hospital; Japan Community Health Care Organization, Osaka Hospital; and Yamaguchi University Hospital from September 2013 to January 2022. Ocular CMV diseases were diagnosed using PCR testing of intraocular fluids, and CMV-viremia cases were diagnosed using CMV pp65 antigenemia assay (C7-HRP). Thirty healthy CMV-seropositive individuals without ocular inflammation were enrolled as healthy controls. Whole blood was collected from 29 CMV-viremia, 19 CMV-AU, 3 CMV-CRN, and 7 CMV-retinitis patients and 30 healthy CMV-seropositive individuals. Peripheral blood mononuclear cells (PBMCs) were isolated from the peripheral blood of 11 CMV-AU patients (mean age 62.3 [30-84] years), 3 CMV-CRN patients (mean age 74.3 [70-82] years), 3 CMV-retinitis patients (mean age 64.3 [60-72] years), and 30 healthy CMV-seropositive individuals (mean age 62.3 [20-84] years) by Ficoll gradient separation and stored in liquid nitrogen until use. A human iris iridectomy specimen was obtained from a CMV-AU patient who underwent glaucoma surgery. This study was conducted in accordance with the Declaration of Helsinki and was approved by the Institutional Review Board of Kyushu University Hospital (Fukuoka, Japan). Written informed consent was received from all participants.

### Deep amplicon sequencing of CMV-UL40 genomic DNA

Genomic DNA was extracted from intraocular fluid and peripheral blood using the QIAamp MinElute Virus Spin Kit and QIAamp DNA Blood Midi Kit (both Qiagen; Venlo, Netherlands), respectively, according to the manufacturer’s instructions. The *UL40* signal peptide region was amplified by PCR using the thermal cycler GeneAmp PCR System 9700 (Applied Biosystems; Waltham, MA, USA) with primers adapted from a previous report (35) for MiSeq sequencing (Illumina; San Diego, CA, USA). The following forward and reverse primers were used: Forward, 5′-TCGTCGGCAGCGTCAGATGTGTATAAGAGACAGCAACAGTCGGCAGAATGAAC-3′ and Reverse, 5′-GTCTCGTGGGCTCGGAGATGTGTATAAGAGACAGCTGGAACACGAGCGGACATA-3′. PCR was performed using KOD Fx polymerase (Toyobo; Osaka, Japan) with the following conditions: one cycle of 94°C for 2 min; 35-60 cycles of 94°C (10 sec), 60°C (30 sec), and 68°C (30 sec), and finally, one cycle at 68°C for 7 min. After confirmation of PCR products by electrophoresis, the products were purified using a QIAquick PCR purification Kit (Qiagen). The Illumina library was prepared from 10 ng of PCR product using a Nextera XT Index Kit v2 (Illumina). Paired-end sequencing (2 × 250 bp) was performed on a MiSeq sequencer (Illumina). After adapter trimming using Cutadapt ver. 3.2, 1,000 reads per sample were randomly sampled using seqtk ver. 1.2. These sampled sequences were then clustered at 100% similarity. Sequence variation was analyzed with a cutoff of 5%, and the frequencies of the nine-mer amino acid sequences of the UL40 signal peptides were quantified. The diversity of sequences was calculated using Simpson's diversity index for each sample (36). The quantification of CMV DNA copies in 28 blood samples from CMV-viremia and 25 ocular fluid samples from ocular CMV disease cases was conducted using the CMV DNA quantification kit (Nihon Techno Service; Ibaraki, Japan) following the manufacturer’s instructions and previous reports (37).

### Establishment of K562-HLA-E transfectant

HLA class-I-deficient K562 cell lines were maintained in RPMI-1640 medium (Sigma-Aldrich; Saint Louis, MO, USA) supplemented with 10% heat-inactivated fetal bovine serum (RPMI-10) and 100 U/ml penicillin-streptomycin (Sigma-Aldrich). The full-length coding region of HLA-E*0103 DNA was cloned into the pMX retroviral vector and co-transfected with VSV-G into Platinum-E packaging cells (kindly provided by Professor T. Kitamura, The Institute of Medical Sciences, The University of Tokyo) (38, 39). K562 cells were transduced with the concentrated viral supernatants, and stable transfectants expressing HLA-E, i.e., K562-HLA-E (K562E cells), were obtained by limiting dilution methods.

### Stabilization of HLA-E on K562E cells

To stabilize HLA-E on the surface of K562E cells, 1 × 10^6^/mL K562E cells were cultured with 200 µM of synthetic peptides consisting of nine-mer amino acids derived from UL40 signal peptides (Eurofins Genomics; Tokyo, Japan) for 15 h at 26°C in RPMI-10. HLA-E expression on K562E cells was analyzed by flow cytometry.

### *In vitro* stimulation of PBMCs

PBMCs were cultured in RPMI-10 with K562E cells either alone or pulsed with 200 μM of synthetic peptides at a ratio of 4:1 for 8 h at 37°C. Anti-CD107a antibody (Biolegend; San Diego, CA, USA) and human recombinant IL-2 (2000 IU/mL; R&D Systems; Minneapolis, MN, USA) were added at the beginning of the assay, and GolgiStop and GolgiPlug (both BD Biosciences; Franklin Lakes, NJ, USA) were added 1 h after the start of the stimulation.

### Antibodies and flow cytometric analysis

The following monoclonal antibodies were used for flow cytometric analysis: APC anti-NKG2C (REA205) (Miltenyi Biotec; Bergisch Gladbach, Germany), Alexa Fluor 700 anti-KIR2DL1/2DS5 (143211) (R&D Systems), PE-Cy7 anti-NKG2A (Z199) (Beckman Coulter; Brea, CA, USA), BV605 anti-KIR2DL2/2DL3/2DS2 (CH-L), V450 anti-IFN-γ (B27) (BD Biosciences), BV650 anti-CD56 (HCD56), BV785 anti-CD3 (SK7), APC/Cyanine7 anti-CD20 (2H7), PE anti-KIR3DL1 (DX-9), BV510 anti-CD107a (H4A3), PE anti-HLA-E (3D12) (Biolegend). Dead cells were excluded using LIVE/DEAD fixable near-IR dead cell stain kit (Thermo Fisher Scientific; Waltham, MA, USA). After staining PBMCs with LIVE/DEAD Fixable Dead Cell Staining kits for 10 min at room temperature, they were washed and stained with mixed monoclonal antibodies for 30 min on ice and then fixed with 1.6% formaldehyde. For intracellular staining of IFN-γ, PBMCs were fixed and permeabilized by incubation with BD Cytofix/Cytoperm (BD Biosciences) for 20 min on ice, washed using BD Perm/Wash (BD Biosciences), stained with anti-IFN-γ antibody for 30 min on ice, and washed and resuspended in PBS containing 2% FBS. PBMCs were analyzed by using a BD FACSAria Fusion Flow Cytometer (BD Biosciences). Flow cytometry data was analyzed using FlowJo software ver. 10 (Tree Star; Ashland, OR, USA).

### *KIR* and *HLA* class I genotyping

Genomic DNA was extracted from whole-blood of 19 CMV-AU, 7 CMV-retinitis, 3 CMV-CRN, and 30 healthy CMV-seropositive individuals using a QIAamp DNA Blood Midi Kit (Qiagen). The sequences of *HLA-A* and *HLA-C* alleles were determined in patients with ocular CMV diseases (19 CMV-AU, 7 CMV-retinitis, 3 CMV-CRN) using the AllType NGS kit (One Lambda, West Hills, CA, USA) as previously described (40). Experimental protocols were carried out following the vendor’s instructions. *HLA* genotypes of patients with CMV-viremia were obtained from clinical records. The presence or absence of 11 variable *Killer cell Immunoglobulin-like Receptor (KIR)* genes was determined in samples from ocular CMV disease cases (11 CMV-AU, 3 CMV-retinitis, 3 CMV-CRN) and 25 healthy CMV-seropositive individuals with the primer sets described in a previous study (41). PCR was conducted using 0.2 μL of Platinum Taq polymerase (Thermo Fisher Scientific) in 12-μL PCR reactions with 3.8 mM of MgCl_2_ and 150 ng of genomic DNA. The PCR conditions were an initial denaturation for 2 min at 95°C, then 10 cycles of 10 sec at 94°C, 40 sec at 65°C, 20 cycles of 20 sec at 94°C, 20 sec at 61°C, and 30 sec at 72°C, and a final 7-min extension at 72°C. All reactions were conducted using the thermal cycler GeneAmp PCR System 9700 (Applied Biosystems). Based on the specificities of the anti-KIR monoclonal antibodies used in the flow cytometry analysis, individuals lacking *2DS2* and *2DS5* were analyzed for 2DLKIR and NKG2C co-expression.

### Immunohistochemistry analysis

Sections (4 μm) prepared from formalin-fixed and paraffin-embedded specimens were deparaffinized with xylene, rehydrated with graded concentrations of ethanol, and rinsed in distilled water. After antigen retrieval with boiling citrate buffer (pH 6.0), the sections were incubated with 5% skim milk for 1 h at room temperature to prevent nonspecific binding and stained with 10 μg/mL anti-HLA-E antibody [MEM-E/02] (Abcam; Cambridge, UK) or IgG from mouse serum (Sigma-Aldrich) overnight at 4°C. Endogenous peroxidase activity was blocked with 0.3% H_2_O_2_/methanol for 30 min at room temperature, and then the sections were incubated with peroxidase-conjugated-anti mouse IgG (Takara; Shiga, Japan) for 30 min at room temperature. Immunostaining was visualized using the AEC peroxidase substrate kit (Vector Laboratories; Burlingame, CA, USA), and tissues were counterstained with hematoxylin. A fluorescent microscope BZ-9000 (Keyence; Osaka, Japan) was used to analyze the slides.

### Statistical analysis

Data were statistically analyzed using GraphPad Prism 9 (GraphPad software; San Diego, CA, USA). The diversity of sequences was compared using a two-tailed t-test. Fisher’s exact test was used for comparing UL40-derived peptide distributions between disease groups. Chi-square test was used for comparing the matching of UL40-derived peptides with host HLA. Nonparametric tests were used to test for statistical significance in the NK cell analyses (two-tailed Mann–Whitney test or Wilcoxon test for paired data). For multiple comparisons, one-way ANOVA multiple comparison tests followed by Dunnett’s multiple comparison tests were used. Pearson correlation was used to analyze correlations between frequencies of signal peptides of UL40 and HLA class I. *P*-values < 0.05 were considered statistically significant.

## Results

### Repertoire of CMV UL40 signal peptide sequences in ocular fluid is distinct from that in peripheral blood

**Figure 1:**
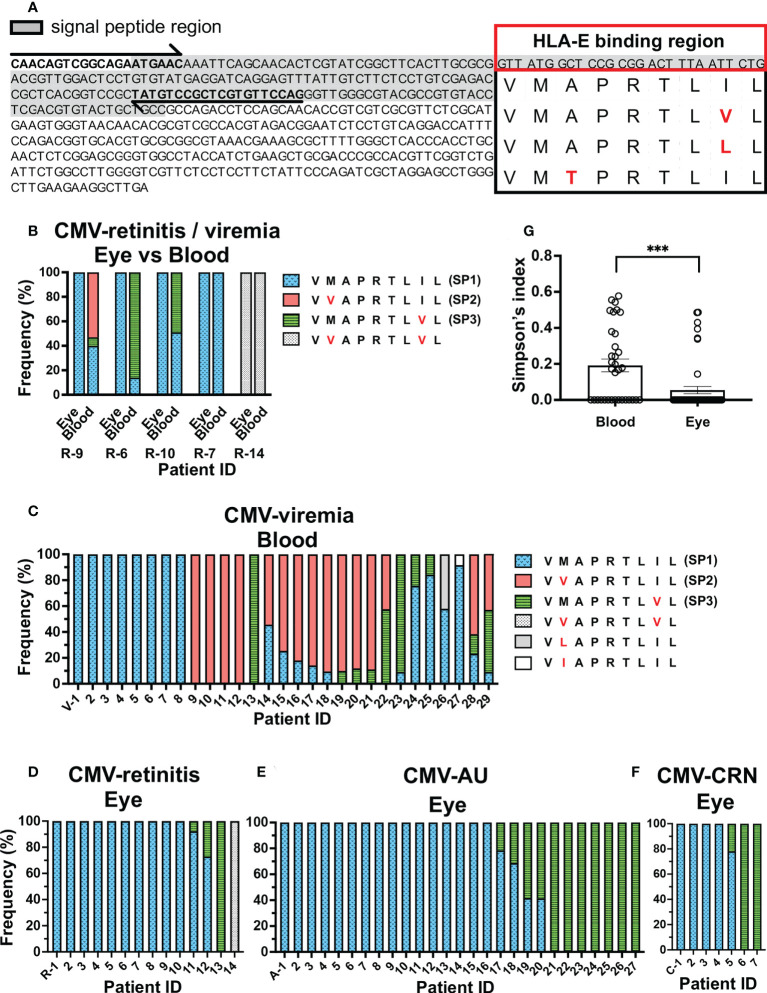
Repertoire of CMV UL40 signal peptide sequences in ocular fluids is distinct from that in peripheral blood. **(A)** Schematic of strategy for detecting UL40 signal sequence region. Full-length *UL40* genetic sequence and the upstream region are shown with primer positions (arrows). Signal peptide region is shaded, and representative HLA-E-binding peptide sequences from previous studies are shown (24, 34, 35). **(B)** Distribution of UL40-encoding signal peptide sequences from five CMV-viremia patients with CMV-retinitis. **(C–F)** Distributions of HCMV UL40-encoding signal peptide sequences identified in peripheral blood of 29 CMV-viremia patients **(C)** and intraocular fluids of 14 CMV-retinitis **(D)**, 27 CMV-AU **(E)**, and 7 CMV-CRN patients **(F)**. X axis is annotated with patient IDs. V-, viremia; R-, retinitis; A-, AU; C-, CRN **(G)** Comparison of UL40 signal peptide sequence diversity between the blood and intraocular fluid using Simpson’s diversity index. Two-tailed t-test was used for statistical analysis. ^***^P = 0.0006.

We first sought to determine whether the distribution of UL40-encoding peptide sequences differed between the ocular fluid and blood from 77 Japanese individuals with ocular CMV diseases and CMV-viremia using deep amplicon sequencing of virus genomic DNA (**Figure 1A**). In five CMV-viremia patients with CMV-retinitis, from whom both blood and ocular fluid were obtained, four different UL40-encoding signal peptide sequences were identified, and sequences detected in the ocular fluid were present in the peripheral blood of all cases (**Figure 1B**). Multiple peptide sequences were observed in the peripheral blood of three patients (R-9, R-6, R-10); however, only V**M**APRTL**I**L (SP1) was identified in their ocular fluid, although this peptide was not the dominant type in their peripheral blood. The presence of multiple UL40 peptide sequences in previous studies was suggested to be due to mixed infections of different HCMV types, which have been observed in 30-40% of blood and urine samples from individuals with systemic HCMV infections (14, 15, 33, 42).

To confirm the differences in UL40 peptide sequences between the peripheral blood and the eye, we extended the study to incorporate 29 peripheral blood samples from patients with CMV-viremia and 14 ocular fluid samples from patients with CMV-retinitis (**Figures 1C, D**). Multiple UL40 peptide sequences were identified in 55% of CMV-viremia blood samples (16/29 cases), whereas they were seen in only 14% of intraocular fluid samples from CMV-retinitis patients (2/14 cases), confirming mixed infections were more frequent in blood than ocular fluid (P = 0.0195). When the most frequent peptide sequences in each sample were assessed, three sequences, V**V**APRTL**I**L (SP2, 45%, 13/29), V**M**APRTL**I**L (SP1, 41%, 12/29), and V**M**APRTL**V**L (SP3, 14%, 4/29), were the major types in blood samples, whereas V**M**APRTL**I**L (SP1, 86%, 12/14 cases) was the dominant type in the ocular fluid of CMV-retinitis. V**M**APRTL**V**L (SP3) and V**V**APRTL**V**L were respectively identified as the most frequent peptide sequence in one patient each. Notably, V**V**APRTL**I**L (SP2) was not identified in the ocular fluid of CMV-retinitis patients (**Figure 1D**).

The limited distribution of UL40 peptide sequences in the ocular fluid of CMV-retinitis patients compared to the peripheral blood of immunocompromised patients prompted us to examine the distributions of UL40 peptide sequences in other ocular CMV diseases which have been identified in non-immunocompromised hosts (CMV-AU and CMV-CRN) by PCR diagnostic methods for ocular fluid recently developed (43–45). We analyzed the UL40 peptide sequences in ocular fluids from 27 CMV-AU and 7 CMV-CRN patients (**Figures 1E, F**). As was observed in the ocular fluid of CMV-retinitis, a single UL40 peptide sequence was identified in most ocular fluid samples from CMV-AU (85%, 23/27 cases) and CMV-CRN (86%, 6/7 cases) patients. Only two UL40 peptide sequences, V**M**APRTL**I**L (SP1) and V**M**APRTL**V**L (SP3), were detected in ocular fluids from CMV-AU and CMV-CRN cases. Notably, neither CMV-AU nor CMV-CRN patient ocular fluids contained UL40 sequences encoding V**V**APRTL**I**L (SP2). In total, multiple UL40 sequences were identified more frequently in blood samples of CMV-viremia compared to ocular fluids of ocular CMV disease cases (19/34 in blood, 7/48 in ocular CMV disease, P = 0.0001). The diversity of UL40 peptide sequences assessed using Simpson’s index indicated that UL40 peptides in the blood of CMV-viremia were more diverse compared to those in the ocular fluid (blood samples; D = 0.19, ocular fluid samples; D = 0.05, P = 0.0006, **Figure 1G**). The limited diversity in ocular fluids was not due to low virus copy numbers, as the ocular fluids of ocular CMV disease cases contained enough amounts of virus DNA (median;1.8x10^6^ copies/mL, 8.9x10^3^-2.7x10^8^ copies/mL) that are comparable to or even higher than the amounts in blood samples from CMV-viremia (median; 7.3x10^5^ copies/mL, 6.9x10^4^-9.6x10^6^ copies/mL). V**M**APRTL**V**L (SP3) tended to be more frequent in the ocular fluids of CMV-AU and CMV-CRN compared to CMV-retinitis cases, although the difference did not reach significance (P = 0.2). Taken together, the evidence shows the limited diversity of UL40 peptide sequences in ocular fluid compared to those in peripheral blood; V**M**APRTL**I**L (SP1) and V**M**APRTL**V**L (SP3) were the two predominant types of UL40 peptide sequences in ocular fluids, regardless of the immune status of the host, and V**V**APRTL**I**L (SP2) was detected in the blood but not the eye.

### Two major UL40-encoding peptides identified in ocular fluids have a strong capacity to inhibit NK cells

**Figure 2:**
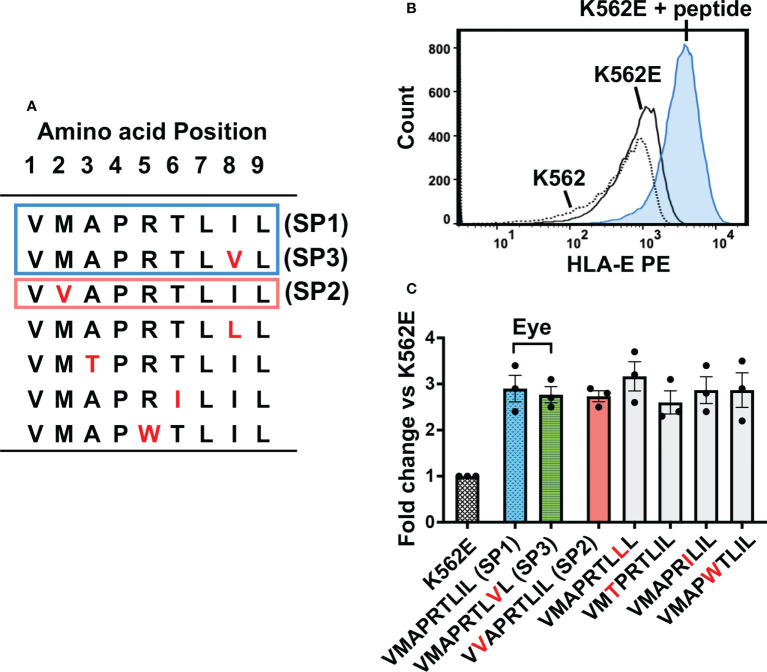
HLA-E expression on K562E cells was comparable between UL40-derived signal peptides. **(A)** UL40-encoding signal peptides tested in this study are shown. The top two peptides (SP1, SP3) (boxed in blue line) were the predominant signal peptide sequences in the intraocular fluid, and the third peptide from the top (SP2) was detected in peripheral blood only (boxed in orange line). Amino acid substitutions compared to SP1 are indicated in red. **(B)** Representative histogram of HLA-E expression on K562, K562E cells cultured in the absence (K562E) or presence of peptides (V**M**APRTL**I**L) (K562E+ peptide). **(C)** HLA-E expression on K562E cells pulsed with each peptide is shown. Data are shown as fold-change normalized to the mean fluorescence intensity (MFI) of HLA-E on K562E cells without peptide pulse. Representative data from three independent experiments are shown as means ± SEM. One-way ANOVA test was used for statistical analysis.

We next sought to assess the functional characteristics of the three major UL40-encoding peptides identified in the Japanese cohorts of CMV-viremia and ocular CMV diseases. When compared with V**M**APRTL**I**L (SP1), the most frequently identified peptide sequence in ocular fluids, V**V**APRTL**I**L (SP2) and V**M**APRTL**V**L (SP3), had amino acid substitutions at positions 2 and 8, respectively (**Figure 2A**). Previous studies reported a greater variety of UL40-encoding peptide sequences that had other amino acid substitutions at positions 3, 5, and 6, all of which have the potential to affect NK cell activation (24, 33, 35, 46). We selected additional UL40 signal peptides with amino acid substitutions distinct from those in previous reports and compared the HLA-E stabilization abilities of all seven UL40-encoding signal peptides (**Figure 2**). The cell surface expression of HLA-E on K562E cells, an HLA-class-I-deficient cell line transfected with HLA-E, was upregulated upon pulsing with UL40-encoding peptides (**Figure 2B**). There were no significant differences in HLA-E expression among the peptides examined (**Figure 2C**).

**Figure 3:**
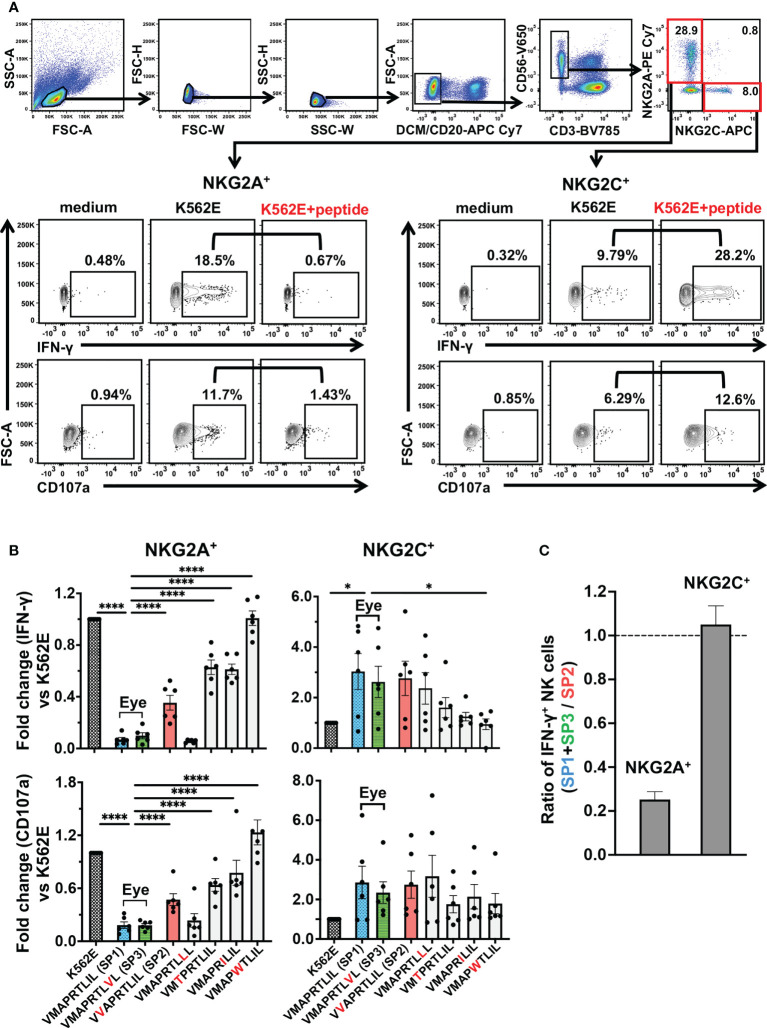
UL40-encoding signal peptides identified in intraocular fluid strongly suppressed NKG2A^+^ NK cells. **(A)** Gating strategy for identification of two NK cell subsets, as determined by NKG2A^+^NKG2C^-^ (NKG2A^+^) and NKG2A^-^NKG2C^+^ (NKG2C^+^), and representative flow cytometry plots of staining for IFN-γ and CD107 in NKG2A^+^ or NKG2C^+^ NK cells cultured in medium alone (medium) or with K562E cells (K562E) or K562E cells pulsed with peptides (V**M**APRTL**I**L) (K562+peptide). **(B)** Inhibitory and activating effects of the seven representative peptides on NKG2A^+^ (two panels on left) and NKG2C^+^ (two panels on right) NK cells were compared. Data are shown as fold-change normalized to the response against unpulsed K562E cells. One-way ANOVA followed by Dunnett’s multiple comparison test. **(C)** Magnitudes of NKG2A-mediated inhibition and NKG2C-mediated activation by SP1 and SP3 were compared with those by SP2. Shown are ratios of anti-SP1 and -SP3 responses of NKG2A^+^ and NKG2C^+^ NK cells to anti-SP2 responses. The means ± SEM are shown. Representative data obtained for healthy CMV-seropositive individuals (n = 6) are shown. ^*^P < 0.05, ^****^P < 0.0001.

We next assessed the inhibitory and activating capacities of the seven peptides on NKG2A^+^ NK cells and NKG2C^+^ NK cells, respectively. As compared to NKG2A, NKG2C recognizes the UL40-derived signal peptides presented by HLA-E with weaker affinity (47). Most NKG2A and NKG2C molecules are expressed exclusively on the NK cell surface, and differential modulation effects have been attributed to UL40-derived signal peptides on NKG2A^+^ and NKG2C^+^ NK cells (24, 33, 35, 48). Both NKG2A^+^ NK cells and NKG2C^+^ NK cells elicited a missing-self response upon co-incubation with K562E cells (**Figure 3A**). Upon co-incubation with K562E cells pulsed with the UL40-encoding peptides, NKG2A^+^ NK cells were inhibited and NKG2C^+^ NK cells were activated in relation to unpulsed K562E-co-incubated cells (**Figure 3A**). Between the two major peptides identified in the eye (V**M**APRTL**I**L; SP1, V**M**APRTL**V**L; SP3), cell there was no significant difference in NKG2A-mediated NK When the NK responses to the two peptides were compared to VVAPRTLIL (SP2), (**Figure 3B**), which was identified in blood but not the eye, V**M**APRTL**I**L (SP1) and V**M**APRTL**V**L (SP3) showed more than three-fold stronger inhibitory capacity towards NKG2A^+^ NK cells, whereas there was no difference in the activation of NKG2C^+^ NK cells (**Figures 3B, C**). We then compared the two peptides V**M**APRTL**I**L (SP1) and V**M**APRTL**V**L (SP3) with the remaining four peptides from previous studies and found that these two peptides had the strongest inhibitory effects on NKG2A^+^ NK cells (**Figure 3B**, two panels on the left). In contrast, NKG2C^+^ NK cell activation effects varied among individuals, and there were no differences between SP1/SP3 and other peptides, except for IFN-γ^+^ NK cells against V**M**AP**W**TL**I**L (**Figure 3B**, two panels on the right). Taken together, these analyses demonstrated that the two major peptides identified in the ocular fluid, V**M**APRTL**I**L (SP1) and V**M**APRTL**V**L (SP3), had a strong capacity to inhibit NK cells.

### NKG2A^+^ NK cells are frequent subsets in CMV-seropositive individuals

**Figure 4:**
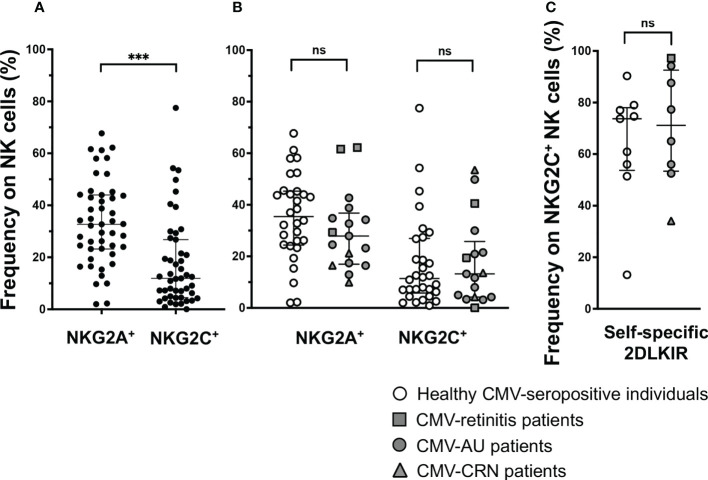
NKG2A^+^ NK cells are frequent in CMV-seropositive individuals. **(A)** Frequency of NKG2A^+^NKG2C^-^ (NKG2A^+^) or NKG2A^-^NKG2C^+^ (NKG2C^+^) NK cells among total NK cells in CMV-seropositive individuals, n = 47. ^***^P < 0.001 by Wilcoxon test. **(B)** Comparison of frequency of NKG2A^+^NKG2C^-^ (NKG2A^+^) or NKG2A^-^NKG2C^+^ (NKG2C^+^) NK cells among total NK cells in healthy CMV-seropositive individuals and patients with ocular CMV diseases (healthy CMV-seropositive individuals, n = 30; CMV-retinitis patients, n = 3; CMV-AU patients, n = 11; CRN patients, n = 3). **(C)** Frequencies of self-specific inhibitory 2DLKIR co-expression on NKG2C^+^ NK cells in healthy CMV-seropositive individuals, n = 9; CMV-retinitis patients, n = 1; CMV-AU patients, n = 6; CMV-CRN patients, n = 1. Two-tailed Mann–Whitney test. Median ± IQR are shown ns, no significant.

NKG2C^+^ NK cells were shown to be expanded in a subset of CMV-seropositive individuals in previous studies and UL40-derived peptides modulate NKG2A^+^ NK cells and NKG2C^+^ NK cells in opposite directions (49). An important question was thus raised as to what degree NK cells can be affected by the significant differences in the UL40 peptide-mediated inhibitory capacities towards NKG2A^+^ NK cells. We thus investigated the frequencies of NKG2A^+^ NK cells and NKG2C^+^ NK cells in CMV-seropositive individuals. Notably, even among CMV-seropositive individuals, the frequency of NKG2A^+^ NK cells was three times higher than that of NKG2C^+^ NK cells (NKG2A median 35.5% vs NKG2C median 11.4%, P < 0.001, **Figure 4A**), and no difference was observed between healthy individuals and CMV disease patients (**Figure 4B**).

Another feature for NKG2C^+^ NK cells is that they highly co-express self-specific inhibitory KIRs, especially KIR2DL1/2/3 (48, 50, 51). Notably, the cognate ligands for KIR2DL1/2/3, HLA-C, are resistant to HCMV US2-mediated HLA class I downregulation; therefore, the activation of NKG2C^+^ NK cells co-expressing self-specific inhibitory KIR2DL1/2/3 is inhibited by the HLA-C expressed on infected cells (50, 51). We then examined how many NKG2C^+^ NK cells co-express self-specific KIR2DL1/2/3 in CMV-seropositive individuals with/without ocular CMV diseases and found them to be co-expressed at around 80%, leaving only around 20% of NKG2C^+^ NK cells free from HLA-C-mediated inhibition (**Figure 4C**). These observations indicate that only a small fraction of NK cells—those expressing NKG2C without self-specific KIR2DL1/2/3 co-expression—are affected by UL40-peptide-mediated activation, and the NK cell population is likely to be more influenced by UL40 peptide-mediated inhibition.

### UL40-encoding signal peptides in ocular fluid are identical to host HLA signal peptides in ocular CMV diseases

**Figure 5:**
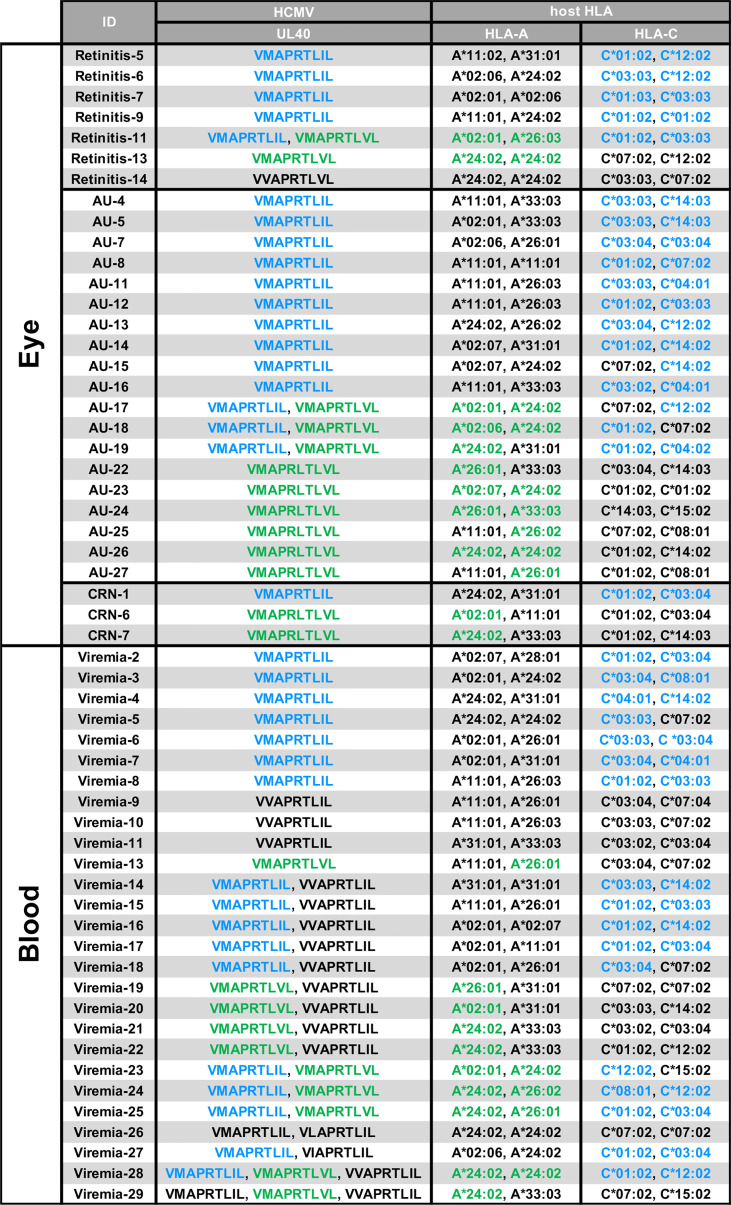
*HLA* genotypes and UL40-encoding signal peptide sequences in blood or intraocular fluid from CMV-viremia and ocular CMV disease patients. HCMV UL40-encoding nine-mer amino acid sequences identified in intraocular fluids or peripheral blood and *HLA-A*/*HLA-C* genotypes of the hosts are shown. *HLA-A* or -*C* alleles with signal peptides identical to HCMV UL40-encoding signal peptides identified in the hosts are indicated in blue (V**M**APRTL**I**L; SP1) and green (V**M**APRTL**V**L; SP3). CMV-viremia, n = 27; CMV-retinitis, n = 7; CMV-AU, n = 19; CMV-CRN, n = 3.

We next assessed potential effects of the UL40 signal peptides identified in this study on HLA-E-restricted CD8^+^T cells (28–30). Notably, the two major UL40-encoding signal peptide sequences in ocular fluid, V**M**APRTL**I**L (SP1) and V**M**APRTL**V**L (SP3), are known to be identical to signal peptides in subsets of HLA-C and HLA-A, respectively (**Supplementary Table 1**) (28). In contrast, V**V**APRTL**I**L (SP2), a major type of UL40-encoding signal peptide in the peripheral blood of CMV-viremia patients, is a motif that is disparate from any HLA class I signal peptide. Previous studies showed that the frequency and response of CMV-specific T cells restricted by HLA-E are comparable to those of conventional T cells, however, the HLA-E-restricted CD8^+^T cell responses against UL40 peptides are absent when the peptides are identical to the host’s HLA class I signal peptides (28–30). We then analyzed the HLA-class I repertoires of hosts with ocular CMV diseases and CMV-viremia and found that all of the UL40 peptide sequences identified in the 28 ocular fluid samples completely matched those of the HLA-A and/or HLA-C of the patients, except for one case, as shown in blue and green in **Figure 5**. In contrast, UL40 peptide sequences in 27 blood samples of CMV-viremia had less similarity to those of the hosts’ HLA, as the UL40 peptide sequences were completely disparate from those of the HLA in four cases, were partially matched in 12 cases, and were completely matched in only 11 cases (P < 0.0001, **Table 1**).

**Table 1:** Number of blood and ocular fluid samples in which UL40 signal peptides matched host HLA.

	Complete match	Partial match	Complete mismatch
Eye	28	0	1
Blood	11	12	4

P < 0.0001.

### HLA-E is expressed on the vascular endothelium of the human eye

**Figure 6:**
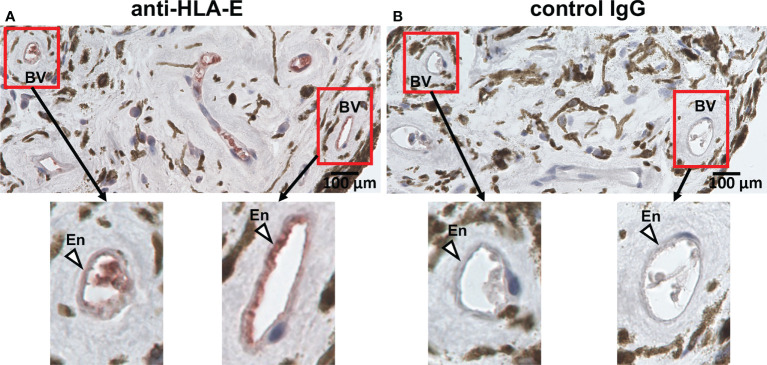
HLA-E is expressed on vascular endothelial cells in iris tissue. Immunohistochemical staining for HLA-E **(A)** and control IgG **(B)** on vascular endothelial cells in iris iridectomy specimens from a CMV-AU patient. **(A)** Endothelial cells positive for HLA-E are stained with red. BV, blood vessel; En, endothelium.

HLA-E is known to be expressed on the vascular endothelium which is considered the primary site of HCMV entry into ocular tissues, thus HLA-E is presumed to be involved in interactions between HCMV and host immune mediators in the eye (9, 52–54). Although a protein homologous to HLA-E, Qa-1b, is expressed in the rodent eye, there has been no clear evidence that HLA-E is expressed in the human eye or vascular endothelium of the ocular tissues (52, 53, 55). We therefore assessed the expression of HLA-E in the human iris and found it to be expressed on the vascular endothelium, suggesting this is a potential site of interaction between HCMV and the host immune system *via* HLA-E preceding the virus’s expansion into the intraocular tissues (**Figure 6**).

### Positive association between HCMV UL40-encoding signal peptides and HLA class I signal peptide frequencies

**Figure 7:**
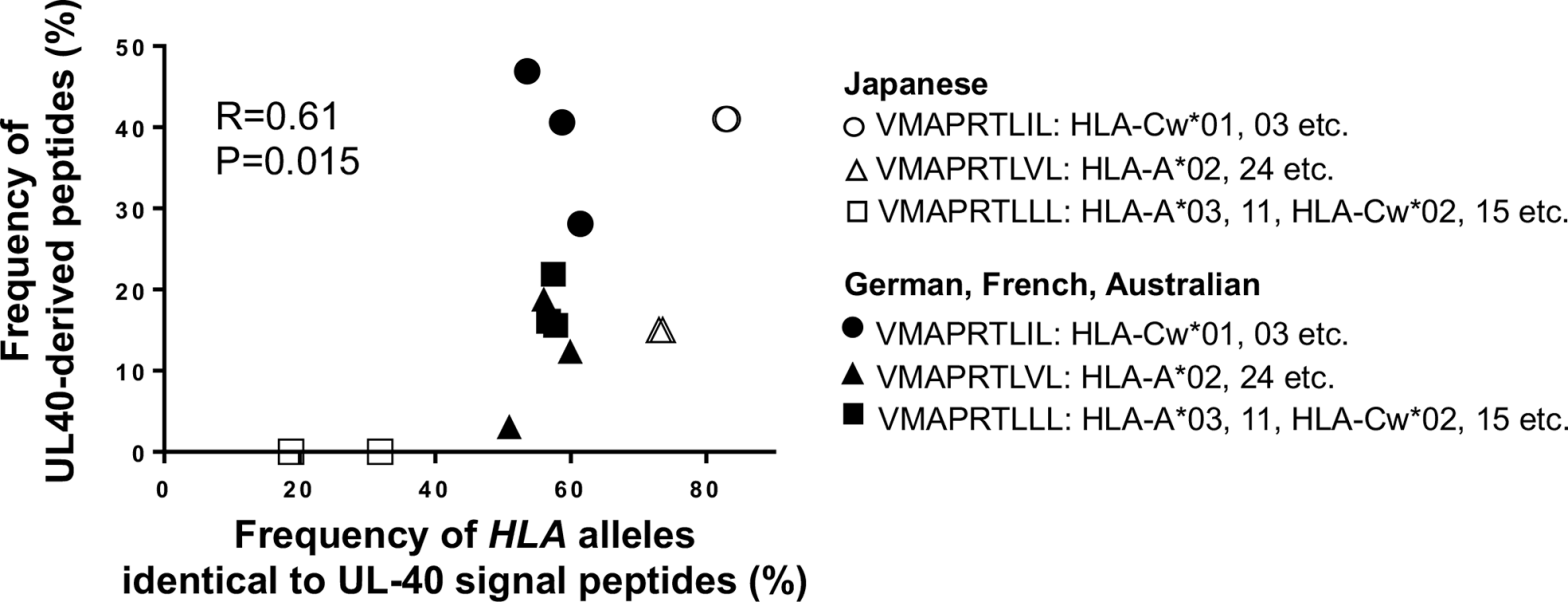
Frequencies of HCMV UL40-encoding signal peptides are positively associated with corresponding *HLA* class I alleles with identical signal peptides among human population groups. Frequencies of UL40-encoding V**M**APRTL**I**L (SP1), V**M**APRTL**V**L (SP3), and V**M**APRTL**L**L and corresponding *HLA* class I allele groups with signal peptides identical to each UL40-encoding signal peptide within the same population were compared. Open circles and closed circles denote Japanese and Caucasian population groups, respectively (Allele Frequency Net Database) (56, 57, 60–62). Pearson correlation coefficient was used in the analysis.

When we compared the UL40-derived peptide sequence repertoire in our cohort with previous reports, we noted that a specific peptide sequence, V**M**APRTL**L**L, was absent in our cohort, whereas it was the second-most frequent UL40 peptide identified in Caucasian CMV-viremia groups, and it is among the three CMV UL40-derived peptides known to be identical to those of classical HLA class I (24, 32, 34, 35). V**M**APRTL**L**L is identical to signal peptides of HLA-A and -C subsets, excluding HLA-A*24, the most frequent HLA-A allotype in the Japanese population (**Supplementary Table 1**) (56, 57). Notably, all three CMV UL40 peptides identical to those of classical HLA class I were described in previous studies conducted on Caucasian populations (24, 32, 34, 35). An important question was then raised as to whether the distributions of CMV UL40 peptides are associated with *HLA* allele group frequencies between populations. To this end, we compared the frequencies of HLA signal peptides corresponding to the UL40 peptides in each population group (**Table 2**). In the Japanese population, *HLA-C* and *-A* allele groups with signal peptides identical to V**M**APRTL**I**L (SP1) and V**M**APRTL**V**L (SP3) occurred at frequencies of 83% and 73%, respectively (**Table 2**) (56, 57). In contrast, the *HLA-A* and *-C* allele groups, with a signal peptide identical to UL40-derived V**M**APRTL**L**L, which was absent in the Japanese cohort, only occurred at a frequency of approximately 20-30% (56, 57). Contrasting with the skewed distributions of the *HLA* allele groups in the Japanese population, the frequency of each of the *HLA* allele groups corresponding to the three UL40 signal peptides in the Caucasian populations was approximately 50% (**Table 2**) (Allele Frequency Net) (60–62). When we compared the frequencies of the three CMV UL40-encoding signal peptides in CMV-viremia samples and the corresponding *HLA* allele groups within the same population, we found them to be positively associated (**Figure 7**, R = 0.61, P = 0.015).

**Table 2:** Frequencies of *HLA-A* and -*C* alleles with signal peptide motifs identical to those of HCMV UL40.

		*HLA-C*	*HLA-A*	*HLA-A, -C*	
Cohort	n	VMAPRTLIL	VMAPRTLVL	VMAPRTLLL	References
**Japanese 1**	5824	**83.1%**	**73.6%**	**31.9%**	(57)
**Japanese 2**	1018	**82.8%**	**73.0%**	**18.7%**	(56)
**South Korean 1**	324	85.3%	54.9%	48.9%	Allele Frequency Net Database*
**South Korean 2**	485	83.4%	49.5%	47.0%	(58)
**Chinese Han 1**	1734	76.2%	50.4%	54.2%	Allele Frequency Net Database*
**Chinese Han 2**	3732	77.3%	50.9%	52.9%	Allele Frequency Net Database*
**Taiwanese** **Han Chinese**	504	73.8%	50.7%	54.0%	(59)
**French**	6094	61.4%	50.9%	57.4%	Allele Frequency Net Database*
**German**	8862	58.7%	59.9%	56.7%	Allele Frequency Net Database*
**Australian**	891	53.6%	56%	57.8%	Allele Frequency Net Database*
**USA Caucasian 1**	1070	63.1%	50.2%	55.1%	(60)
**USA Caucasian 2**	1242890	60.4%	49.6%	57.4%	(61)

* (62) The bold values highlighted the Japanese data.

## Discussion

To the best of our knowledge, this is the first study comparing UL40 signal peptide sequences between infected local tissues and the peripheral blood of CMV disease cases in the context of their similarity to host HLA and the immunological consequences. UL40 signal peptide sequences were more diverse and multiple sequences were more common in blood compared to the intraocular fluid of CMV-viremia cases. Three UL40 peptides, V**M**APRTL**I**L (SP1), V**V**APRTL**I**L (SP2), and V**M**APRTL**V**L (SP3), were the dominant types in the peripheral blood. In contrast, only two of the three UL40 peptides, V**M**APRTL**I**L (SP1) and V**M**APRTL**V**L (SP3), were observed in the majority of intraocular fluid specimens from CMV-retinitis patients (in which one of the peptides was observed in each patient), and V**V**APRTL**I**L (SP2) was not present in their ocular fluid. The distributions of UL40 peptides in intraocular fluids were similar between the various ocular CMV disease cases, and greater differences in UL40 peptide repertoires existed between the eye and blood.

Based on the peptide profiles identified in this study, we assessed the differences in their immunosuppressive capacity against NK cells. The two major peptide sequences observed in the ocular fluid, V**M**APRTL**I**L (SP1) and V**M**APRTL**V**L (SP3), both possess a strong inhibitory capacity, whereas the peptide found only in blood, V**V**APRTL**I**L (SP2), was more than three-fold weaker. Previous studies showed amino acid residues at positions 3, 5, and 8 determine the NK cell inhibitory capacity mediated by NKG2A (24, 33). Our study inferred that position 2 is also a critical determinant in NK cell inhibition. There was no difference in NK cell inhibitory or activating capacity between V**M**APRTL**I**L (SP1) and V**M**APRTL**V**L (SP3), confirming a previous study on the binding affinity of HLA-E/UL40 peptide complexes for NKG2A, although the amino acid at position 8 has been proposed to be involved in the interaction between NK cells and HLA-E (24, 46). Another question addressed in this study was whether the differential effects of the UL40 peptides are attributable to their recognition by the activating NKG2C receptor. As demonstrated in **Figure 3**, the peptides studied did not differentiate NK cell effector functions in NKG2C^+^NK cells, indicating that UL40 variation mainly affects NK cell inhibition through NKG2A.

Having demonstrated how UL40 variants differentially modulate NK cell effector function, we considered the role of T cell recognition of the HLA-E/UL40 peptide complex. Previous studies have described the HLA-E presentation of various pathogen-derived peptides along with self-peptides originating from the MHC class Ia signal sequence, indicating that HLA-E has substantial roles in immune surveillance and distinguishing self from non-self and that this is a conserved system in humans and rodents (25, 31, 54, 63, 64). Studies on murine CMV infections have suggested that anti-CMV responses by Qa-1-restricted CD8^+^T cells are comparable to those by conventional T cells and compensate for the virus’s evasion of conventional CD8^+^T cells (65). Studies in humans have described an interesting phenomenon in which the frequency and response of CMV-specific T cells restricted by HLA-E are comparable to those of conventional T cells when the HCMV UL40 peptide sequence does not match any of the host’s HLA class I signal peptides (28, 30). Conversely, previous studies have described a lack of HLA-E-restricted CD8^+^T cell responses against UL40 peptides when the peptides are identical to the host’s HLA class I signal peptides. These studies have attributed the observation to the negative selection of CD8^+^T cells specific for self-peptides in the thymus, as simulated in mouse Qa-1 systems (28, 30, 31). Outstanding questions on whether the CMV UL40-derived peptides in hosts with CMV diseases are associated with the host’s HLA remained, and in our study, we discovered that HCMV in ocular fluids carried the UL40 variant that matched the leader peptide sequence of at least one of the host’s HLA class I allotypes in 96% (27/28 cases) of ocular CMV disease versus only 40% (11/27 cases) of CMV-viremia cases.

**Figure 8:**
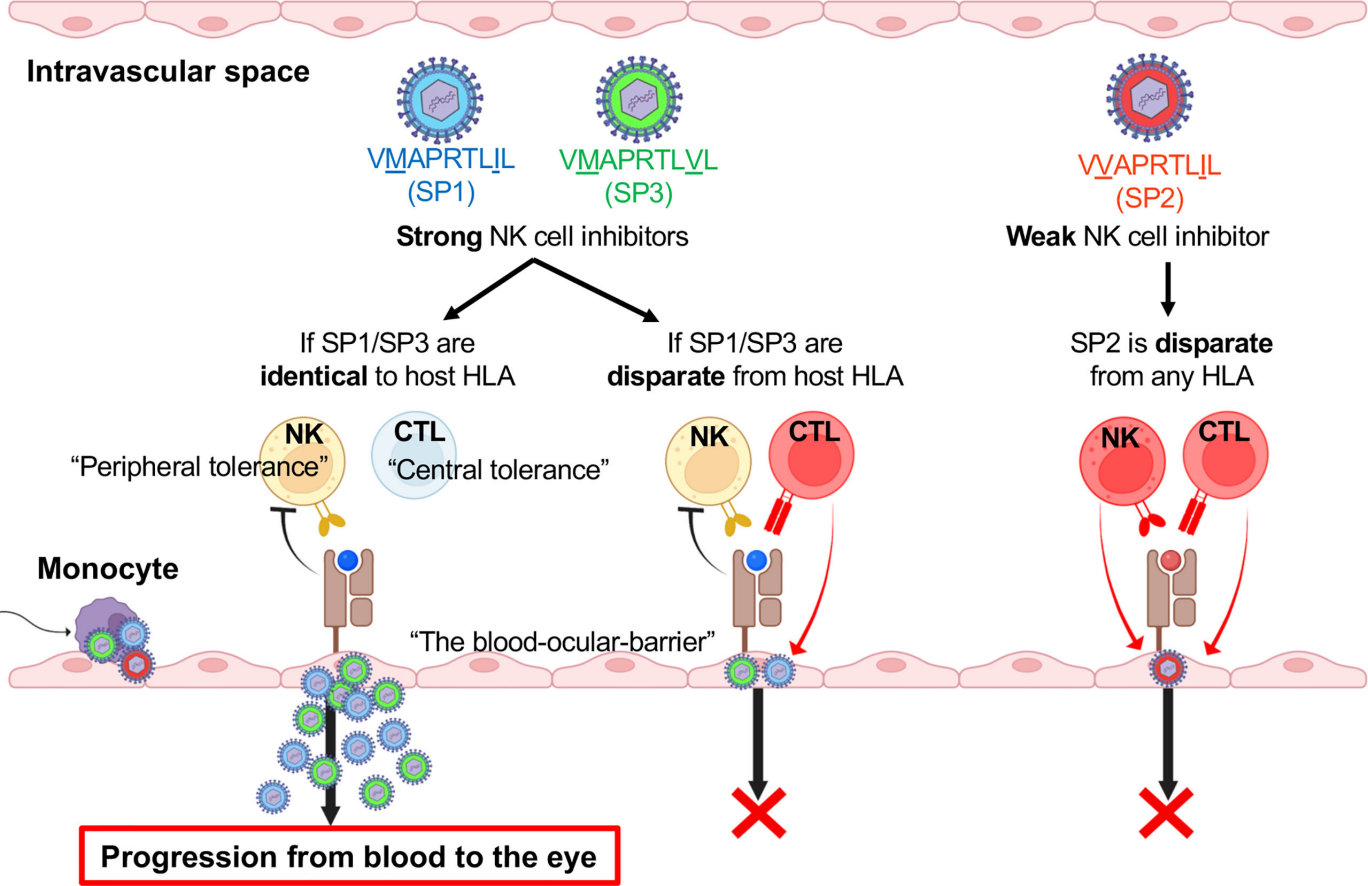
HLA-E-mediated immune escape is dependent on signal peptide polymorphisms of HCMV UL40 and host HLA at the blood–eye interface. Schematic overview of the hypothesis for HCMV progression into the eye involving virus utilization of central and peripheral tolerance mechanisms.

Taken together, the evidence implicates two conceivable immunological effects of the clinical HCMV strains in ocular fluids carrying HLA leader peptide-matched UL40 peptides: (1) to counteract the downregulation of HLA-E by providing an alternative source of peptides to present to HLA-E and to induce strong inhibitory signaling through NKG2A on NK cells and (2) to escape HLA-E-restricted CD8^+^T cells through the exploitation of self-tolerance against host *HLA* alleles. The multi-faceted strategy by which HCMV evades innate and adaptive cytotoxic lymphocytes is likely one that involves the utilization of a central tolerance mechanism as well as the induction of virus-tolerant peripheral NK cells at the blood-ocular interface. **Figure 8** depicts our current hypothesis of how combinations of HCMV UL40 signal peptides and those of the host’s HLA result in viral escape from innate and adaptive immunity.

Mixed infections with multiple HCMV types are commonly observed in blood and urine samples from individuals with systemic HCMV infections (14, 15, 33, 42). Given that HCMV mutates during *in vitro* culture to adapt to the host cells, the question is whether HCMV produces new variants over the course of an infection (66). This point was investigated in previous studies in which the appearance of new sequences was not evident when HCMV types were sampled from the same individual across different time points during an infection (33, 67). However, mutations occurring in different tissues could be an additional factor creating differences between the UL40 peptide sequence repertoires of the blood and eye.

Various viruses are known to have evolved a wide range of ways to escape host immune attack (68–70). HCMV has adopted multiple means of immune evasion, but the strategy centering on mutating the UL40 genome segment to mimic classical HLA class I expression is unique. This approach is, however, a contingent of the virus’s ability to adapt to geographic variations in *HLA* allele distributions. From this aspect, this study has highlighted the need to investigate HCMV strains in conjunction with HLA class I allotypes in population groups. Geographic clusters have been described for other herpesvirus genomes, such as those of herpes simplex virus and varicella zoster virus; however, the vast lack of genomic information for Asian HCMV types has not enabled such studies for HCMV (32). From this perspective, this is the first study to analyze potential differences in HCMV UL40-encoding peptides between Asian and Caucasian population groups. Among the various UL40 peptides, three signal peptides are identical to those of HLA-A or HLA-C (28). Based on an assessment of the distribution of *HLA* alleles in Japan, the majority of the population carries *HLA* alleles corresponding to V**M**APRTL**I**L (SP1) and V**M**APRTL**V**L (SP3), which were the major UL40 types in our cohort (**Table 2** and **Figure 7**) (56, 57). Other East Asian population groups also carry *HLA* alleles corresponding to V**M**APRTL**I**L (SP1) at higher frequencies compared to Caucasian population groups (**Table 2**). In contrast, only half of the Caucasian population groups studied carry *HLA* allele groups matching V**M**APRTL**I**L (SP1), V**M**APRTL**V**L (SP3), and V**M**APRTL**L**L (Allele Frequency Net Database) (60–62). This led to the unexpected and thought-provoking finding that the frequencies of UL40-encoding signal peptide sequences may be associated with those *HLA* alleles with corresponding leader peptide sequences in the population groups (**Figure 7**). These results infer that HCMV strains that have escaped host immunity through the evasion of HLA-E-restricted unconventional CD8^+^ T-cell recognition are those that have adapted to each human population group and are capable of maintaining a latent infection. Indeed, viral adaptations to immune selective pressure by HLA class I-restricted CD8^+^ T cells are a recognized feature of HIV (71, 72).

Related to this finding is the identification of a new disease entity: CMV-AU. Contrary to our previous understanding that clinical manifestations of CMV are limited to immunocompromised hosts, CMV-AU occurs in immunocompetent hosts and is now recognized as the most frequent cause of infectious uveitis in East Asian populations (73, 74). The incidence of CMV-AU is 2.8- to 34-times higher in East Asian compared to Caucasian population groups, much greater than the difference in the incidence of latent HCMV infections (80-90% vs. 50% in Asian vs. Caucasian population groups) (74–80). Although the etiology of CMV-AU is as yet unclear, differences in the genetic backgrounds of both the virus and host have been highlighted as possible factors in the pathogenesis (81, 82).

The clinical significance of UL40 peptide variation was highlighted in a recent report that revealed a particular UL40 peptide in peripheral blood is associated with frequent CMV-reactivation after lung transplantation (33). The correlation between disease incidence and the frequencies of UL40 peptide sequences matching *HLA* allele leader peptide sequences warrants investigations into the distribution of CMV variants and immunological studies to better understand the mechanisms of CMV diseases. The number of refractory CMV-retinitis cases with hematologic malignancies has been increasing in recent years (83). The potential use of UL40 peptide types and host HLA as predictors of the risk of retinitis development is one subject for future study (2–6). Moreover, these findings suggest routes for the development of new therapeutics for this difficult-to-treat disease entity. As demonstrated in this study, UL40 peptides in all ocular CMV diseases confer the same immune-escape properties, and whether other viral genes are involved in the differentiation of various ocular CMV diseases is as yet unclear. Additional studies on virus genomes and gene functional analyses are warranted to further understand the mechanisms of various ocular CMV diseases.

## Data availability statement

UL40 signal sequence data are available at the NCBI (PRJNA869040).

## Ethics statement

The studies involving human participants were reviewed and approved by Institutional Review Board of Kyushu University Hospital. The patients/participants provided their written informed consent to participate in this study.

## Author contributions

MS: Conceptualization, design of study, execution of experiments, acquisition of data, analysis and interpretation of data, sample collection, and drafting of the manuscript. NY: Conceptualization, design of study, sample collection, analysis and interpretation of data, and drafting and critical revision of the manuscript, obtaining funding, and study supervision. DM: design of study, execution of experiments, acquisition of data, analysis and interpretation of data, and drafting and critical revision of the manuscript. KS: design of study, execution of experiments, critical revision of the manuscript. S-SK and YO: design of study, execution of experiments, acquisition of data, analysis and interpretation of data. TK, RY, HM, YMo, EH, AN, AT, MH, NO, KA: sample collection and critical revision of the manuscript. MA: analysis and interpretation of data. SY, TI, AH, YMu, KF: sample collection. KM, KT, MY: critical revision of the manuscript, MP, WY: obtaining funding and critical revision of the manuscript, K-HS: obtaining funding, study supervision, and critical revision of the manuscript. All authors agree to be accountable for the content of the work.

## Funding

This work was supported by the Japan Society for the Promotion of Science (JSPS), grant numbers JP17H07303, JP18K09467, JP21K09723, and JP20H05873; the Japan Agency for Medical Research and Development (AMED), grant numbers JP20jk0210029 and 22ama121037; the National Institute of Allergy and Infectious Diseases (NIAID), grant number 66947; Bursky Center for Human Immunology and Immunotherapy Programs; and Rheumatic Diseases Research Resources-based Center, National Institute of Arthritis and Musculoskeletal and Skin Diseases, NIH, grant number P30AR073752.

## Acknowledgments

We thank Toshio Kitamura for supplying Platinum-E packaging cells; Daniel Geraghty, Akiko Ishitani, Gabriel Gonzalez, Satoko Nakano for their valuable comments and discussion; and Yuka Matsutani, Fumiyo Morikawa, Masayo Eto, Tantri Lestari, The Research Support Centre, Kyushu University Graduate School of Medical Sciences, for their technical assistance. We appreciate the suggestions provided during the manuscript review. The authors would also like to thank Insight Editing London for editing the manuscript prior to submission.

## Conflict of interest

The authors declare that the research was conducted in the absence of any commercial or financial relationships that could be construed as potential conflict of interest.

## Publisher’s note

All claims expressed in this article are solely those of the authors and do not necessarily represent those of their affiliated organizations, or those of the publisher, the editors and the reviewers. Any product that may be evaluated in this article, or claim that may be made by its manufacturer, is not guaranteed or endorsed by the publisher.
