# Corrigendum: Intraocular human cytomegaloviruses of ocular diseases are distinct from those of viremia and are capable of escaping from innate and adaptive immunity by exploiting HLA-E-mediated peripheral and central tolerance

**DOI:** 10.3389/fimmu.2022.1124440

**Published:** 2023-01-05

**Authors:** Mariko Shirane, Nobuyo Yawata, Daisuke Motooka, Kensuke Shibata, Seik-Soon Khor, Yosuke Omae, Toshikatsu Kaburaki, Ryoji Yanai, Hisashi Mashimo, Satoshi Yamana, Takako Ito, Akira Hayashida, Yasuo Mori, Akihiko Numata, Yusuke Murakami, Kohta Fujiwara, Nobuyuki Ohguro, Mayumi Hosogai, Masato Akiyama, Eiichi Hasegawa, Michael Paley, Atsunobu Takeda, Katsumi Maenaka, Koichi Akashi, Wayne M. Yokoyama, Katsushi Tokunaga, Makoto Yawata, Koh-Hei Sonoda

**Affiliations:** ^1^ Department of Ophthalmology, Kyushu University, Fukuoka, Japan; ^2^ Department of Ocular Pathology and Imaging Science, Kyushu University, Fukuoka, Japan; ^3^ Ocular inflammation and Immunology, Singapore Eye Research Institute, Singapore, Singapore; ^4^ Ophthalmology and Visual Sciences Academic Clinical Program, Duke-NUS Medical School, Singapore, Singapore; ^5^ Department of Infection Metagenomics, Research Institute for Microbial Diseases, Osaka University, Osaka, Japan; ^6^ Integrated Frontier Research for Medical Science Division, Institute for Open and Transdisciplinary Research Initiatives (OTRI), Osaka University, Osaka, Japan; ^7^ Department of Microbiology and Immunology, Graduate School of Medicine, Yamaguchi University, Yamaguchi, Japan; ^8^ Department of Molecular Immunology, Research Institute for Microbial Diseases, Osaka University, Osaka, Japan; ^9^ Genome Medical Science Project, National Center for Global Health and Medicine, Tokyo, Japan; ^10^ Department of Ophthalmology, The University of Tokyo Hospital, Tokyo, Japan; ^11^ Department of Ophthalmology, Jichi Medical University Saitama Medical Center, Saitama, Japan; ^12^ Department of Ophthalmology, Yamaguchi University Graduate School of Medicine, Yamaguchi, Japan; ^13^ Department of Ophthalmology, Japan Community Health Care Organization Hospital, Osaka, Japan; ^14^ Department of Medicine and Biosystemic Science, Kyushu University Graduate School of Medical Science, Fukuoka, Japan; ^15^ Department of Ophthalmology, Gunma University Graduate School of Medicine, Gunma, Japan; ^16^ Department of Medicine, Washington University School of Medicine, St. Louis, MO, United States; ^17^ Center for Research and Education on Drug Discovery, Faculty of Pharmaceutical Sciences, Hokkaido University, Sapporo, Japan; ^18^ Laboratory of Biomolecular Science, Faculty of Pharmaceutical Sciences, Hokkaido University, Sapporo, Japan; ^19^ Global Station for Biosurfaces and Drug Discovery, Hokkaido University, Sapporo, Japan; ^20^ Bursky Center for Human Immunology and Immunotherapy Programs, Washington University, St. Louis, MO, United States; ^21^ Singapore Institute for Clinical Sciences (SICS), Agency for Science, Technology and Research, ASTAR, Singapore, Singapore; ^22^ Department of Pediatrics, Yong Loo Lin School of Medicine, National University of Singapore, Singapore, Singapore; ^23^ Department of Pediatrics, National University Health System, Singapore, Singapore; ^24^ Immunology Programme, Life Sciences Institute, National University of Singapore, Singapore, Singapore; ^25^ National University Singapore Medicine Immunology Translational Research Programme, National University of Singapore, Singapore, Singapore; ^26^ International Research Center for Medical Sciences, Kumamoto University, Kumamoto, Japan

**Keywords:** human cytomegalovirus, HLA-E, UL40, natural killer cells, HLA class I, NKG2A, CMV viremia, CMV retinitis

In the published article, there were errors. In the original article, there was an error in the description of the Reverse primer sequence in Materials and methods.

A correction has been made to **Materials and methods**, “*Deep amplicon sequencing of CMV-UL40 genomic DNA”*.

This sentence previously stated:

“The following forward and reverse primers were used: Forward, 5′-TCGTCGGCAGCGTCAGATGTGTATAAGAGACAGCAACAGTCGGCAGAATGAAC-3′ and Reverse, 5′-GTCTCGTGGGCTCGGAGATGTGTATAAGAGACAGCTGGAACACGACGCATA-3’.”

The corrected sentence appears below:

“The following forward and reverse primers were used: Forward, 5′-TCGTCGGCAGCGTCAGATGTGTATAAGAGACAGCAACAGTCGGCAGAATGAAC-3′ and Reverse, 5′-GTCTCGTGGGCTCGGAGATGTGTATAAGAGACAGCTGGAACACGAGCGGACATA-3’.”

In the original article, there was another error in the description of the concentration of the anti-HLA-E antibody in **Materials and methods**.

A correction has been made to **Materials and methods**, “*Immunohistochemistry analysis”*.

This sentence previously stated:

“After antigen retrieval with boiling citrate buffer (pH 6.0), the sections were incubated with 5% skim milk for 1 h at room temperature to prevent nonspecific binding and stained with 10 μL/mL anti-HLA-E antibody [MEM-E/02] (Abcam; Cambridge, UK) or IgG from mouse serum (Sigma-Aldrich) overnight at 4°C.”

The corrected sentence appears below:

“After antigen retrieval with boiling citrate buffer (pH 6.0), the sections were incubated with 5% skim milk for 1 h at room temperature to prevent nonspecific binding and stained with 10 μg/mL anti-HLA-E antibody [MEM-E/02] (Abcam; Cambridge, UK) or IgG from mouse serum (Sigma-Aldrich) overnight at 4°C.”

The authors apologize for these errors and state that these do not change the scientific conclusions of the article in any way. The original article has been updated.

