# Supplementary material for: Intraocular human cytomegaloviruses of ocular diseases are distinct from those of viremia and are capable of escaping from innate and adaptive immunity by exploiting HLA-E-mediated peripheral and central tolerance
Source: Front Immunol. 2022 Oct 19;13:1008220. doi: 10.3389/fimmu.2022.1008220 (PMC9626817; doi:10.3389/fimmu.2022.1008220)
Supplement: Supplementary file 1 [file Table_1.docx]

Supplementary Table 1 HLA-A and HLA-C allotypes which have signal peptide motifs identical to those of HCMV UL40

| Signal peptide sequences | HLA class I allotypes |
| --- | --- |
| VMAPRTL**I**L (SP1) | HLA-Cw*01, 03, 04, 05, 06, 08, 12, 14, 16, 1702 |
| VMAPRTL**V**L (SP3) | HLA-A*02, 23, 24, 25, 26, 3402, 43, 66, 69 |
| VMAPRTL**L**L | HLA-A*01, 03, 11, 29, 30, 31, 32, 33, 36, 74, HLA-Cw*02, 15 |
